# Anti-Inflammatory, Anti-Diabetic, and Anti-Alzheimer’s Effects of Prenylated Flavonoids from Okinawa Propolis: An Investigation by Experimental and Computational Studies

**DOI:** 10.3390/molecules23102479

**Published:** 2018-09-27

**Authors:** Md Shahinozzaman, Nozomi Taira, Takahiro Ishii, Mohammad A. Halim, Md Amzad Hossain, Shinkichi Tawata

**Affiliations:** 1 The United Graduate School of Agricultural Sciences, Kagoshima University, Korimoto 1-21-24, Kagoshima 890-0065, Japan; mshahin81@gmail.com (M.S.); ishiit@agr.u-ryukyu.ac.jp (T.I.); amzad@agr.u-ryukyu.ac.jp (M.A.H.); 2 PAK Research Center, University of the Ryukyus, Okinawa 903-0213, Japan; 3 Department of Bioscience and Biotechnology, Faculty of Agriculture, University of the Ryukyus, Senbaru 1, Nishihara-cho, Okinawa 903-0213, Japan; taira5935@gmail.com; 4 Division of Computer Aided Drug Design, The Red-Green Research Centre, 218 Elephant Road, Dhaka 1205, Bangladesh; mahalim@grc-bd.org; 5 Subtropical Field Science Center, University of the Ryukyus, Senbaru 1, Nishihara-cho, Okinawa 903-0213, Japan

**Keywords:** Okinawa propolis, prenylated flavonoids, inflammation, α-glucosidase, acetylcholinesterase, molecular docking, density functional theory, p21-activated kinase 1

## Abstract

Okinawa propolis (OP) and its major ingredients were reported to have anti-cancer effects and lifespan-extending effects on *Caenorhabditis elegans* through inactivation of the oncogenic kinase, p21-activated kinase 1 (PAK1). Herein, five prenylated flavonoids from OP, nymphaeol-A (NA), nymphaeol-B (NB), nymphaeol-C (NC), isonymphaeol-B (INB), and 3′-geranyl-naringenin (GN), were evaluated for their anti-inflammatory, anti-diabetic, and anti-Alzheimer’s effects using in vitro techniques. They showed significant anti-inflammatory effects through inhibition of albumin denaturation (half maximal inhibitory concentration (IC_50_) values of 0.26–1.02 µM), nitrite accumulation (IC_50_ values of 2.4–7.0 µM), and cyclooxygenase-2 (COX-2) activity (IC_50_ values of 11.74–24.03 µM). They also strongly suppressed in vitro α-glucosidase enzyme activity with IC_50_ values of 3.77–5.66 µM. However, only INB and NA inhibited acetylcholinesterase significantly compared to the standard drug donepezil, with IC_50_ values of 7.23 and 7.77 µM, respectively. Molecular docking results indicated that OP compounds have good binding affinity to the α-glucosidase and acetylcholinesterase proteins, making non-bonded interactions with their active residues and surrounding allosteric residues. In addition, none of the compounds violated Lipinski’s rule of five and showed notable toxicity parameters. Density functional theory (DFT)-based global reactivity descriptors demonstrated their high reactive nature along with the kinetic stability. In conclusion, this combined study suggests that OP components might be beneficial in the treatment of inflammation, type 2 diabetes mellitus, and Alzheimer’s disease.

## 1. Introduction

Acute inflammation is thought be a good defense strategy to remove injurious stimuli and to initiate the healing process in the body. Chronic inflammation (CI), by contrast, is considered unfavorable, as it is involved in many diseases and disorders through its prolonged, dysregulated, and maladaptive effects [1]. Inflammation and its acute responses are well defined, whereas little is known on the development and progression of CI [2]. During CI, increasing numbers of macrophage cells at inflammatory sites play an important role in the defense system, but produce several toxins themselves including reactive oxygen or nitrogen species (ROS or RNS). They together stimulate CI, inducing cycloxygenase-2 (COX-2), inflammatory cytokines (tumor necrosis factor alpha (TNFα), interleukin 1 (IL-1), and IL-6), chemokines (IL-8 and C–X–C chemokine receptor 4 (CXCR4)), and pro-inflammatory transcription factors (nuclear factor kappa B (NF-κB)) [3]. CI was demonstrated as a common feature in the natural course of diabetes, and the inflammatory mediators correlate with diabetic incidence and prevalence [4]. Inflammatory responses contribute to type 2 diabetes (T2D) occurrence by causing insulin resistance, and, in turn, they are intensified in the presence of hyperglycemia and promote long-term diabetic complications [4]. Both inflammation and T2D trigger the age-related neurodegenerative disorder, Alzheimer’s disease (AD) [5,6], which is often termed as type 3 diabetes due to its pathophysiological similarities with T2D [7]. Individuals with T2D have nearly a twofold higher risk of AD than non-diabetic individuals [8]. Importantly, AD patients also have elevated insulin levels, which play a vital role in developing neuropathological hallmarks of AD through increasing amyloid beta (Aβ) accumulation and tau phosphorylation in the brain [9,10,11]. Hence, targeting inflammatory pathways could be a significant strategy for treating diabetes [12,13], and subsequently, for diminishing AD onset and progression [14,15,16].

Propolis is a complex resinous material collected by honeybees, which has been used as a potent herbal and dietary supplement since ancient times of human civilization. Its pharmacological properties were extensively studied in several fields including inflammation, hypertension, diabetic complications, infectious diseases, microbial contaminations, cancer, and so on. Depending on surrounding vegetation, propolis collected from different regions shows differences in its major ingredients. For example, New Zealand propolis contains caffeic acid phenethyl ester (CAPE), Brazilian propolis contains artepillin C, and Taiwan propolis contains propolin G [17]. However, the major ingredients of Okinawa propolis (OP) are a variety of prenylated flavonoids which originate from *Macaranga tanarius* plant [18]. These components were found to have anti-oxidant, anti-bacterial, and anti-angiogenic effects [17,19]. Previously, our group worked with OP and reported that it directly inhibits the major oncogenic kinase, p21-activated kinase 1 (PAK1), and hence, it is suggested to be utilized as a potent herbal drug for treating cancer, hyperpigmentation, and for extending the lifespan [17]. In another work, nymphaeol A (NA) and nymphaeol C (NC), two major compounds of OP, were demonstrated to have strong selectivity in their inhibitory effects on PAK1 compared to that on other oncogenic kinases [20]. PAK1 is responsible for a variety of human diseases/disorders and it is currently considered to be a major therapeutic target for the treatment of cancer, diabetes, hypertension, and neurodegenerative disorders [21]. Suppressive effects of OP and its ingredients on PAK1 activities, therefore, encouraged us to evaluate their anti-inflammatory, anti-diabetic, and anti-Alzheimer’s effects. Herein, we used both in vitro techniques and computational approaches, which precisely explained the pharmacological effects of OP compounds and their drug likeness, as well as their toxicological and quantum chemical properties. This combined effort could effectively be used as a potential guideline for expanding OP as a safe natural drug for treating inflammation, diabetes, and neurodegenerative disorders.

## 2. Results and Discussion

### 2.1. Anti-Inflammatory Effects

**Figure 1:**
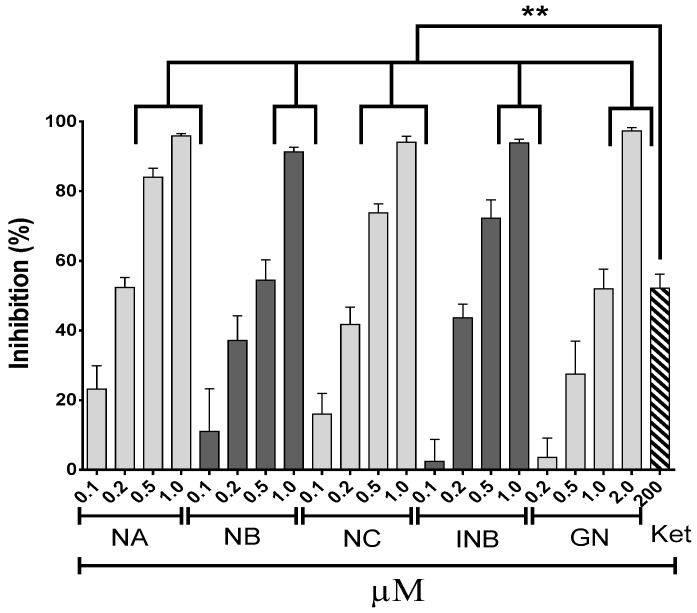
Inhibitory effects of Okinawa propolis (OP) compounds on albumin denaturation in vitro. Ketorolac (Ket) was used as a control, and asterisks indicate significant differences compared to the positive control (** *p* < 0.01).

**Figure 2:**
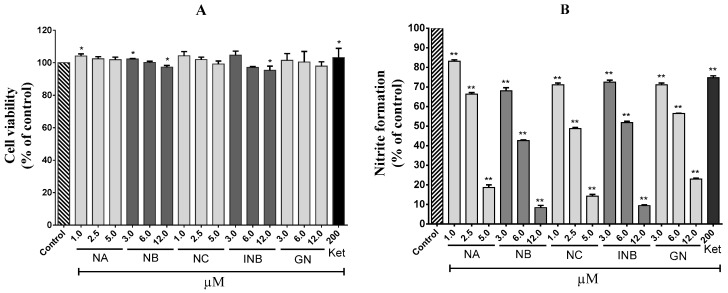
Effects of different compounds on RAW 264.7 cell viability (**A**) and on nitrite formation (**B**) in lipopolysaccharide (LPS)-stimulated RAW 264.7 cells. Ketorolac (Ket) was used as a standard drug. Asterisks indicate significant differences compared to the control (* *p* < 0.05; ** *p* < 0.01).

**Figure 3:**
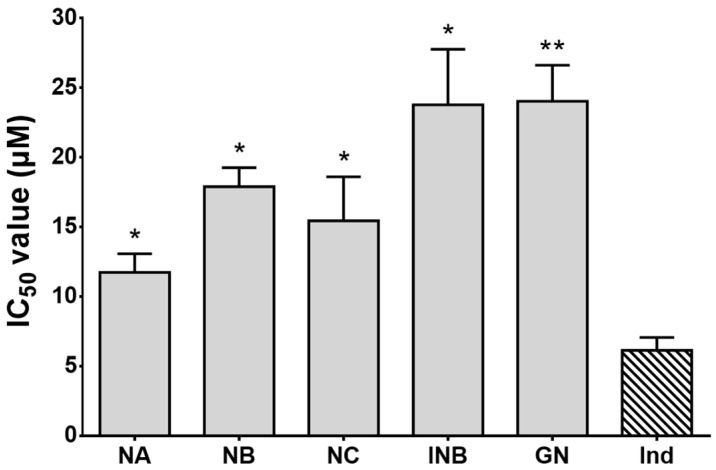
Cycloxygenase-2 (COX-2) inhibitory effects of Okinawa propolis (OP) compounds. Results are expressed as means ± standard error (SE) of three repeated experiments. Indomethacin (Ind) was used as a standard inhibitor. Asterisks indicate significant differences compared to the positive control (* *p* < 0.05; ** *p* < 0.01).

As shown in Figure 1, OP compounds inhibited albumin denaturation in a dose-dependent manner. They all showed strong inhibitory effects, and the results were found to be significant compared to ketorolac, a non-steroidal anti-inflammatory drug (NSAID). Out of all compounds, NA showed the best effects followed by NC, isonymphaeol-B (INB), nymphaeol-B (NB), and 3′-geranyl-naringenin (GN). The half maximal inhibitory concentration (IC_50_) values for NA, NC, INB, NB, and GN were 0.26, 0.37, 0.42, 0.54, and 1.02 µM, respectively. However, ketorolac at a 200 µM concentration inhibited only 52% albumin denaturation. Since cellular proteins are denatured due to inflammation, the drugs showing inhibitory action against protein denaturation seem to be effective for treating inflammation [22]. For this purpose, OP compounds might be a suitable candidate for further studies. Next, they were tested on lipopolysaccharide (LPS)-stimulated RAW 264.7 cells and were found to have stronger effects on nitrite accumulation in culture supernatants. Firstly, all the compounds were assessed for their cytotoxicity on RAW 264.7 cells through the 3-(4,5-dimethylthiazol-2-yl)-2,5-diphenyltetrazolium bromide (MTT) assay. Most of the compounds at tested concentrations did not inhibit cell proliferation; rather, they induced cell proliferation to some extent. However, NB and INB at 12 µM concentration significantly inhibited cell proliferation but with a slight deviation (approximately 3–5%) from the control group. Hence, NB and INB at 12 µM concentration were used in the subsequent nitrite assay. The standard drug, ketorolac, at 200 μM concentration did not show cytotoxicity on RAW 264.7 cells, and showed a significant effect on nitrite formation in the supernatant with 26% inhibition compared to the cells treated only with LPS. However, OP compounds exerted stronger and concentration-dependent inhibitory effects on nitrite formation than that of ketorolac (Figure 2). NC showed the highest inhibitory effects with an IC_50_ value 2.4 µM followed by NA, NB, INB, and GN (whose IC_50_ values were 3.2, 5.4, 6.2, and 7.0 µM, respectively) (Table 1). They also inhibited COX-2 activity dose-dependently in LPS-induced RAW 264.7 cells, and the IC_50_ values for NA, NB, NC, INB, and GN were 11.74, 17.90, 15.45, 23.78, and 24.03 µM, respectively (Figure 3). In LPS-treated macrophage cells, two major pro-inflammatory mediators, nitric oxide (NO) and prostaglandin E2 (PGE_2_) are produced from l-arginine and arachidonic acid, respectively, and these reactions are catalyzed by transcriptional activation of the inducible nitric oxide synthase (*i*NOS) and COX-2 genes, respectively [23,24]. Importantly, *i*NOS and COX-2 are downstream signaling components of PAK1 [25,26]. Hence, it can be assumed here that OP compounds firstly inhibit PAK1, and subsequently, suppress the catalytic activities of *i*NOS and COX-2. Taken together, OP components could protect cellular proteins and reduce the formation of NO and PGE_2_ in inflamed tissues, and therefore, they could be utilized as herbal drugs for treating inflammation and other related disorders.

### 2.2. α-Glucosidase and Acetylcholinesterase (AChE) Inhibitory Effects

**Figure 4:**
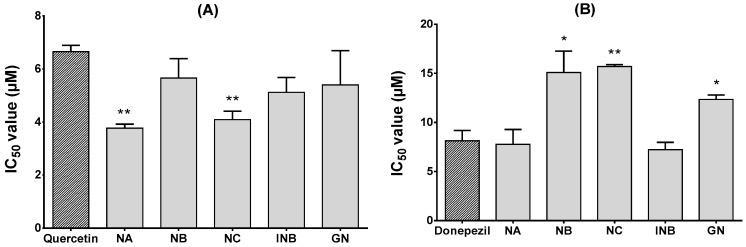
The α-glucosidase (**A**) and acetylcholinesterase (**B**) inhibitory effects of different Okinawa propolis (OP) compounds. Data represent means ± SE of three experiments. Asterisks indicate significant differences compared to the positive control (* *p* < 0.05; ** *p* < 0.01).

The anti-diabetic effects of OP compounds were evaluated by testing their yeast α-glucosidase inhibitory activities. The enzyme α-glucosidase, secreted in the small intestine, is essential for carbohydrate digestion and the subsequent increase in postprandial blood glucose levels. An abnormal increase in postprandial blood glucose level is thought to be a major reason for T2D progression [27]. Hence, α-glucosidase inhibitors are recommended as an oral anti-diabetic drug which can retard carbohydrate degradation, thus delaying and reducing the level of postprandial hyperglycemia [27]. Apart from these factors, α-glucosidase inhibitors might also provide other health benefits such as moderating plasma triglyceride levels with cardiovascular disorders and hypertension risks through reducing glucose toxicity and improved insulin response [28]. All compounds tested showed stronger inhibition of in vitro α-glucosidase activity than the positive control quercetin (IC_50_ = 6.65 µM). NA and NC inhibited significantly with IC_50_ values of 3.77 and 4.09 µM, respectively (Figure 4), whereas the IC_50_ values for NB, INB, and GN were 5.66, 5.12, and 5.40 µM, respectively. These findings indicate that OP compounds can act as anti-diabetic drugs for treating T2D.

Like α-glucosidase inhibition, all compounds showed suppressive effects on electric eel AChE enzyme activity when tested in vitro (Figure 4). INB and NA inhibited AChE strongly compared to the standard drug donepezil (IC_50_ = 8.13 µM), with IC_50_ values of 7.23 and 7.77 µM, respectively. GN, NB, and NC also inhibited AChE, but with lower effects than donepezil. The IC_50_ values for GN, NB, and NC were 12.34, 15.09, and 15.70 µM, respectively. AChE is localized in synaptic gaps of the central and peripheral nervous system and is responsible for the breakdown of acetylcholine (ACh). Thus, AChE terminates nerve impulses through the loss of basal forebrain cholinergic neurons and by reducing the level of the neurotransmitter ACh, which is characteristic of AD as a chronic neurodegenerative disorder [29,30]. Inhibition of AChE increases cholinergic functions, and hence, it is the therapeutic target for not only managing AD, but also for moderating other disorders such as myasthenia gravis, glaucoma, and Lewy body dementia [30]. Since synthetic AChE inhibitors have some adverse effects, such as hepatotoxicity and gastrointestinal complaints, OP compounds reported here could be utilized successfully as natural-product-derived drugs for treating AD and other cholinergic dysfunctions.

### 2.3. Molecular Docking Study

**Figure 5:**
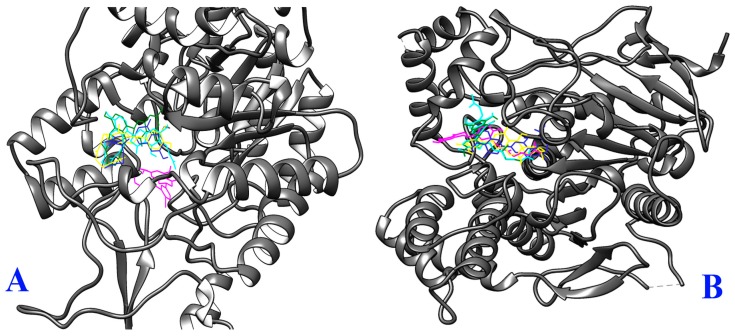
Binding orientations of Okinawa propolis (OP) compounds within α-glucosidase (Protein Data Bank identifier (PDB ID): 3A4A) and acetylcholinesterase (PDB ID: 4EY7) protein receptors. (**A**) Binding orientation within 3A4A (nymphaeol-A (NA) = yellow; nymphaeol-B (NB) = green; nymphaeol-C (NC) = cyan; isonymphaeol-B (INB) = blue; 3′-geranyl-naringenin (GN) = magenta), and (**B**) binding orientation within 4EY7 (NA = yellow; NB = green; NC = cyan; INB = blue; GN = magenta).

**Figure 6:**
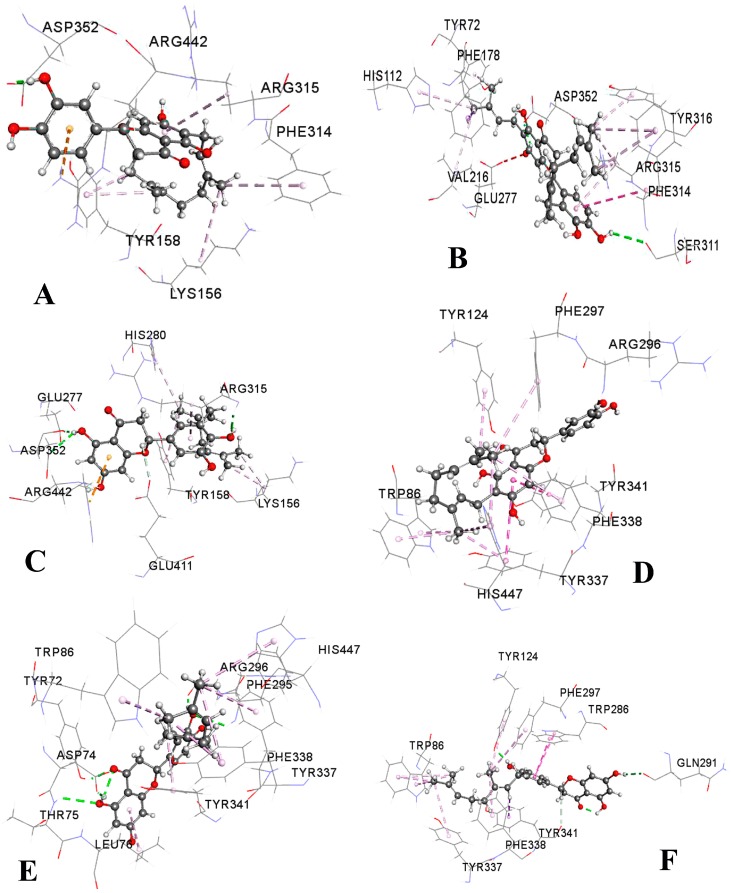
Non-bonded interactions of the best-docked compounds with α-glucosidase (Protein Data Bank identifier (PDB ID): 3A4A) and acetylcholinesterase (PDB ID: 4EY7) proteins. (**A**) Nymphaeol-A (NA) with 3A4A, (**B**) nymphaeol-C (NC) with 3A4A, (**C**) isonymphaeol-B (INB) with 3A4A, (**D**) NA with 4EY7, (**E**) nymphaeol-B (NB) with 4EY7, and (**F**) 3′-geranyl-naringenin (GN) with 4EY7.

To understand the mechanisms of α-glucosidase and AChE inhibition by OP compounds, as well as the binding mode inside the binding pocket of the enzymes, and to confirm the experimental results, molecular docking simulations were performed in this study. All the compounds were docked to the crystal structure of isomaltase (Protein Data Bank identifier (PDB ID): 3A4A) which has strong similarity to α-glucosidase. They showed promising binding affinities with variable free binding energies ranging from −7.1 to −9.9 kcal/mol, clarifying their wide-spectrum structural and functional features. Except for GN, all other compounds could be embedded into the same binding pocket of 3A4A (Figure 5A). The results in terms of binding energy, non-bonded interactions, and bond distance are presented in Table 2. According to the binding affinities, the compounds could be ranked as NC ˃ NA ˃ INB ˃ NB ˃ GN. However, the experimental results revealed their rank as NA ˃ NC ˃ INB ˃ NB ˃ GN. Both the docking simulation and the experimental results demonstrated NA and NC as the most active α-glucosidase inhibitors. The three-dimensional structures of non-bonded interactions of the three best OP compounds with 3A4A are presented in Figure 6. All compounds showed different types of non-bonded interactions such as hydrogen bonding, hydrophobic bonding, and electrostatic bonding with several residues of the active site or close to the active site. NA and NC showed one hydrogen-bond interaction with the active site residue Asp 352, whereas INB formed two hydrogen bonds with two different catalytic residues, Asp 352 and Glu 277. On the other hand, NB and GN did not interact with the active-site residues, but they interacted with other residues close to the catalytic cleft.

When the compounds were docked with AChE (PDB ID: 4EY7), they showed almost similar binding energies ranging from −11.0 to −11.5 kcal/mol. NC and INB showed identical binding affinity (−11.0 kcal/mol), but the highest binding affinity was demonstrated by NA, followed by GN and NB. The free binding energies for NA, GN, and NB were −11.5, −11.3, and −11.2 kcal/mol, respectively (Table 2). On the other hand, NA was found to be the second highest AChE inhibitor after INB through in vitro experiments. However, all compounds could bind to the same pocket of AChE with similar orientation (Figure 5B). Non-bonded interactions of all compounds with different protein residues are presented in Table 2. In AChE, Trp 86 is termed as the choline-binding site residue which interacts with the ligand through hydrophobic interactions [31]. Surprisingly, all tested compounds showed hydrophobic interactions with Trp 86 with π–σ, π–π stacked, or π–alkyl bond formation. NB and NC interacted with AChE via the formation of two and three hydrogen bonds, respectively, with the catalytic residues Tyr 72 and Phe 295. These two residues were found to be involved in donepezil binding (PDB ID: 4EY7). INB interacted with one catalytic residue Phe 295 along with another residue Asp 74 which is close to the catalytic pocket. However, NA and GN did not interact with any catalytic residues, but they interacted with the close residues of catalytic site. NA interacted with AChE only through hydrophobic bonds, whereas GN formed two hydrogen bonds with AChE backbone residues Gln 291 and Tyr 124 (Figure 6).

The free binding energies, binding modes, and interactions of OP compounds with α-glucosidase and AChE protein calculated from this in silico study demonstrate that these compounds might show promising interactions with the target proteins, and thus, could slow carbohydrate breakdown in the small intestine and the catabolism of ACh in synaptic cleft. In accordance with the experimental findings, the in silico studies indicate that major components of OP could act as herbal drugs for treating T2D and AD, although further investigations are warranted to explore their in-depth mechanism of action.

### 2.4. Pharmacokinetic and Toxicological Properties

Pharmacokinetic properties (PKs) are thought to be important in drug development, since they determine the characteristic features for a successful oral drug which is promptly and completely absorbed from the gastrointestinal tract, distributed to the site of action, metabolized well, and eliminated in a suitable manner without causing any detrimental effects. That is why many drugs under clinical trial fail to commercialize due to having poor PKs. PKs depend on the chemical descriptors of the molecule. Computational predictions are currently used in drug discovery programs to explore absorption, distribution, metabolism, excretion, and toxicity (ADMET) profiling of new drug candidates with the clear aim of selecting only drug-like compounds having optimal PKs [32]. The Molinspiration online property calculation toolkit was used here to screen ADMET properties of OP compounds as future drug candidates based on Lipinski’s rule of five [33]. The results are presented in Table 3. According to this rule, orally administered drugs should have a molecular weight of ˂500 amu, a LogP value ≤ 5, five or fewer hydrogen-bond donor sites, and ten or fewer hydrogen-bond acceptor sites. Drug candidates violating one of the above rules may have problems with bioavailability. Interestingly, none of the OP compounds violate these rules, and hence, all may have good oral bioavailability.

Additionally, based on Veber’s rule, orally bioavailable drugs should have the number of rotatable bonds below or equal to 10, and topological polar surface area (TPSA) values ≤140 Å^2^ [34]. The number of rotatable bonds is thought be a good descriptor for suitable drugs, whereas TPSA is involved in passive molecular transport of drugs through membranes. All OP compounds have a number of rotatable bonds lower than 10 and TPSA values lower than 140 Å^2^. According to Zhao et al. [35], the calculated percentage of absorption for OP compounds ranged from 72.01–78.99%.

Toxicological properties of OP components were also predicted using the admetSAR server (Table 4). The results demonstrated that none of the compounds posed a risk of Ames toxicity and carcinogenicity. However, all of the compounds were found to be weak inhibitors for the human ether-a-go-go-related gene (hERG) and showed weak rat acute toxicity with a median lethal dose (LD_50_) of 3.1399 mol/kg. As per the predicted acute oral toxicity values, all the compounds lie in “class III”. Compounds of this class have LD_50_ values greater than 500 mg/kg but less than 5000 mg/kg and are generally considered suitable from a druggable point of view [36]. Thus, OP compounds are qualified for use as promising drugs with good oral bioavailability and safety features.

### 2.5. Density Functional Theory (DFT)-Based Computations

DFT-based computation has, indeed, the potential of becoming a very important tool in computer-aided drug design (CAD) with the aim of developing new drugs for new targets, and thus, for medicinal chemistry [37]. DFT-based scoring functions for molecular descriptors might play an important role in quantitative structure–activity relationships (QSARs), pharmacology, genomics, drug design, toxicology, proteomics, analytical chemistry, virtual screening, and so forth. Global and local descriptors of DFT may also significantly correlate computed and experimental drug activities. In this investigation, we carried out DFT calculations of OP compounds to understand the structural features, reactive nature, and sites of the compounds essential for biological activities, which enable the design of new drugs with potential effects, as well as attractive materials with applications in industry. The results are presented in Table 5, which includes changes in electronic energy, enthalpy, and Gibbs free energy, as well as dipole moment, highest occupied molecular orbital energy (εHOMO), lowest unoccupied molecular orbital energy (εLUMO), hardness, and softness. Greater negative values of thermodynamic properties indicate improved thermodynamic features of the compounds. Dipole moment is the indicator of drug–receptor interaction, which facilitates hydrogen-bond interactions [38]. All OP compounds show comparatively higher dipole moments ranging from 2.235–5.228 Debey, and NC has the highest dipole moment (5.228 Debey) which agrees with its experimental interaction with the target receptors used in this study.

According to the frontier molecular orbital theory, HOMO and LUMO energies play important roles in the chemical reactivity and kinetic stability features of drugs [39]. The energy gap of HOMO–LUMO also determines the hardness and softness of drugs. Large gaps denote a hard molecule and small gaps signify a soft molecule [40]. The reactivity of drugs increases with their softness. All the OP compounds show relatively low HOMO–LUMO energy gaps ranging from 0.1599–0.1684 Hartree and high softness ranging from 11.8715–12.4564 Hartree, demonstrating that they have a strong reactive nature with their targets. Frontier orbitals are displayed in Figure S1, which reflect the localization pattern of frontier molecular orbitals in OP compounds. Their localization in different positions demonstrate their structural and functional diversity. However, in all compounds, the frontier molecular orbitals are located mostly in aromatic moieties not in the geranyl side chain, which clarifies the active role of aromatic moieties during the interaction with their target receptors.

## 3. Materials and Methods

### 3.1. Chemicals and Reagents

Chemicals and reagents used in this study were of analytical grade. Dulbecco’s modified Eagle medium (DMEM), casein, Griess reagents (0.1% *N*-(1-naphtyl)-ethylenediamine and 1% sulfanilamide in 5% orthophosphoric acid), 70% perchloric acid, MTT, α-glucosidase, and acetylcholinesterase were purchased from Sigma-Aldrich (Saint Louis, MO, USA). Fetal bovine serum (FBS) was purchased from HyClone, Victoria, Australia. DMEM without phenol red was collected from Thermo Fisher Scientific (Waltham, Massachusetts, USA). Chicken egg albumin, trypsin, and penicillin/streptomycin were purchased from Funakoshi Co. Ltd. (Tokyo, Japan). All other chemicals used were obtained from either Wako Pure Chemical Industries Ltd. (Osaka, Japan) or Kanto Chemical Co., Inc. (Tokyo, Japan).

### 3.2. Sample Collection and Purification of Compounds

**Figure 7:**
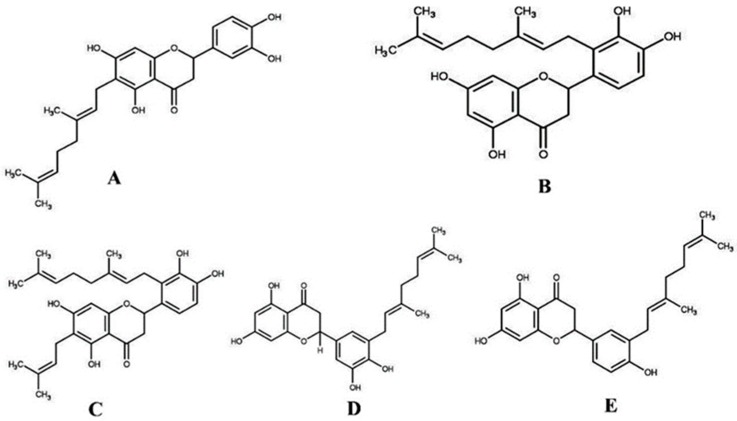
Chemical structures of the purified compounds from Okinawa propolis. (**A**) Nymphaeol-A (NA), (**B**) nymphaeol-B (NB), (**C**) nymphaeol-C (NC), (**D**) isonymphaeol-B (INB), and (**E**) 3′-geranyl-naringenin (GN).

Propolis collected from the Okinawa Yoho bee farm (Okinawa, Japan) was dried at 45 °C and then grounded with a mortar and pestle to make powder. Then, 1 g of powder was extracted with 50 mL of ethanol by sonicating for 3 h and shaking for 24 h at 25 °C (Bio-Shaker, BR-300LF, Taitec Corporation, Tokyo, Japan). Extraction was collected by filtering with Whatman filter paper and centrifuged several times at 4000 rpm for 5 min. Finally, the clear supernatant was dried with a rotavapor apparatus (BUCHI, Flawil, Switzerland) under reduced pressure, and a yellow-colored dried crude extract was obtained (yield: 32%). Crude extract was dissolved in ethanol at 30 mg/mL concentration and subjected to high-performance liquid chromatography (HPLC; Shimadzu, Kyoto, Japan) using a semipreparative C18 column (COSMOSIL 5C18-AR-II 250 mm × 10 mm, Nacalai Tesque, Kyoto, Japan). Distilled water (solvent A) and acetonitrile (solvent B) were used as mobile phases with a flow rate of 3.0 mL/min. HPLC conditions were as follows: 0–30 min, isocratic conditions 80% solvent B; 30.01–38 min, linear gradient 80–100% solvent B; 38.01–40 min, linear gradient 100–10% solvent B; 40 min, stop. Five compounds, NA, NB, NC, INB, and GN (Figure 7), were purified and their chemical structures were confirmed based on the comparison of their physical data to that of published data [18].

### 3.3. Albumin Denaturation Inhibition Assay

Albumin denaturation inhibition effects of the compounds were confirmed through a previously described method [41]. Egg albumin was used in this experiment, and the reaction mixture contained 0.1 mL of egg albumin, 0.7 mL of phosphate-buffered saline (PBS), and varying concentrations of different compounds (0.5 mL). Mili-Q water (0.5 mL) instead of the compounds was used in the negative control. The reaction mixtures were incubated at 37 °C for 15 min and heated at 70 °C for 5 min. The absorbance was measured at 660 nm after cooling at room temperature. Ketorolac was used as a standard drug. Percentage of albumin denaturation inhibition was calculated according to the following formula:

Percentage of inhibition = (1 − (A/B)) × 100,

where A is the absorbance of the test sample, and B is the absorbance of the negative control.

### 3.4. Cell Viability Assay

RAW 264.7 cell viability was evaluated through the MTT assay [42]. Cells were seeded in a 24-well plate at a density of 2 × 10^5^ cells/well and incubated with the OP compounds for 24 h. Ketroloac (200 μM) was also assessed to determine its cytotoxicity on RAW 264.7 cells. After incubation, the supernatant was removed, and the cells were washed with PBS. Aliquots of 100 μL of MTT solution (0.5 mg/mL in PBS) were added to each well, and the plate was incubated at 37 °C for 3 h. Afterwards, dimethyl sulfoxide (400 μL) was added to each well, and the plate was shaken for 10 min at room temperature to dissolve the formazan crystals. The absorbance was measured at 570 nm wavelength using a microplate reader, and the cell viability was calculated from the absorbance of the treated versus untreated control cells.

### 3.5. Nitrite Inhibition Assay

As an indicator of NO synthesis, nitrite amount was measured in the supernatant of LPS-stimulated RAW 264.7 cells. Cells were plated on a 24-well plate with a density of 2 × 10^5^ cells/well. After seeding, cells were treated with LPS (500 ng/mL final concentration) and varying concentrations of the test compounds for 24 h. Ketorolac (200 μM) was used as a standard drug. Then, 100 μL of culture supernatant from each well was mixed with an equal volume of Griess reagent, and incubated at room temperature for 10 min. The formation of an azo compound was measured spectrophotometrically at 550 nm wavelength. Finally, nitrite concentration was determined from a standard curve of sodium nitrite, and nitrite inhibition percentages of test compounds and the reference drug were calculated based on the amount of nitrite produced by the control cells (treated only with LPS).

### 3.6. Cell-Based Assay for COX-2 Inhibition

This assay was carried out according to the method described by Stanikunaite et al. [43] with some modifications. In brief, RAW 264.7 cells (2 × 10^5^ cells/well) were cultured in a 24-well plate in DMEM supplemented with 10% FBS and incubated at 37 °C for 24 h for seeding. After seeding, cells were washed with PBS (400 µL) and treated with 500 ng/mL LPS for the next 24 h to induce the production of COX-2. Then, the induced cells were washed thoroughly with PBS to remove LPS completely and treated with different concentrations of test compounds for 4 h. Arachidonic acid (300 µM) was added and the cells were further incubated for 30 min. Next, the PGE_2_ levels were determined in the culture supernatant using a PGE_2_ ELISA kit (Cayman Chemical Co., Ann Arbor, MI, USA) according to the manufacturer’s instructions. COX-2 enzyme activity was determined by the conversion of exogenous arachidonic acid to PGE_2_, and was expressed as the percentage of negative control (without test compounds). Finally, IC_50_ values were calculated for all the tested compounds. Indomethacin was used as a positive control.

### 3.7. α-Glucosidase Inhibition Assay

The α-glucosidase assay was performed with slight modifications of a previous method [44]. In short, 15 µL of the test sample at various concentrations was added to 140 µL of enzyme solution (0.0073 U/mL of α-glucosidase in 0.05 M sodium phosphate buffer containing 100 mM NaCl) in a 96-well plate. Reaction mixtures were incubated at 37 °C for 15 min. Then, 25 µL of 0.7 mM *p*-nitrophenyl-*α*-d-glucopyranoside (PNPG) solution in 0.05 M sodium phosphate buffer (pH 6.8) was added to each well. The increase in absorption at 405 nm wavelength due to the hydrolysis of PNPG by α-glucosidase was monitored continuously with a microplate reader.

### 3.8. Acetylcholinesterase Inhibition Assay

The AChE inhibitory properties of the compounds were determined using a 96-well microplate colorimetric method [45] as described by Ellman et al. [46]. Donepezil was used as a positive control. Each sample was tested in triplicate, and the percentage of inhibition was determined as follows:

AChE inhibition (%) = (1 − (sample reaction rate/blank reaction rate)) × 100.

### 3.9. Molecular Docking

#### 3.9.1. Preparation of Receptors

The crystal structures of target proteins were retrieved from the Research Collaboratory for Structural Bioinformatics (RCSB) Protein Data Bank (http://www.rcsb.org). Isomaltase from *Saccharomyces cerevisiae* co-crystallized with maltose (PDB ID: 3A4A) was used here as the α-glucosidase protein since it shows 85% similarity to yeast α-glucosidase (MAL12) through homology modeling [47]. On the other hand, acetylcholinesterase protein co-crystalized with donepezil (PDB ID: 4EY7) was used for docking calculations. Water molecules, heteroatoms, and ligands were removed from the crystal structure of proteins using Discovery Studio 4.5 (Accelrys, San Diego, CA, USA). Polar hydrogen atoms were also added with Discovery Studio 4.5. The energy minimization of receptors was then carried out with Swiss-PdbViewer 4.1 (Swiss Institute of Bioinformatics, Lausanne, Switzerland).

#### 3.9.2. Preparation of Ligands

Chemical structures of all compounds were drawn by Marvin Sketch (ChemAxon, Budapest, Hungary), and then, were converted to three-dimensional format using ChemBioDraw Ultra 12.0 (CambridgeSoft, Cambridge, MA 02140, USA). Geometry optimization was done with Gaussian09 software [48] by density functional theory (DFT) at the B3LYP/6–31G (d,p) level of theory. Finally, all the compounds were saved as pdb (Protein data bank) format for further docking simulations.

#### 3.9.3. Docking Simulation

AutoDock Vina (The Scripps Research Institute, La Jolla, CA, USA) was used here for the molecular docking simulations. As an open source program, it is widely used for molecular docking, which significantly improves the accuracy of the binding mode predictions compared to AutoDock 4 [49]. Depending on the binding mode of co-crystalized ligands, active site residues of the proteins were determined using Discovery Studio 4.5 (Accelrys, San Diego, CA, USA), and then, the grid box size was defined accordingly. In the α-glucosidase protein, the dimensions (Å) of the grid box were 45.24, 29.91, and 25.00, and the center (*x*, *y*, *z*) of the grid box was 33.06, −7.65, 18.63. Similarly, in the acetylcholinesterase protein, the dimensions (Å) of the grid box were 49.96, 45.69, and 25.00, and the center (*x*, *y*, *z*) of the grid box was −7.34, −44.19, 30.87.

#### 3.9.4. Analysis and Visualization of Docking Results

After the docking simulation, the docked pose with the highest negative value was selected as the best for the corresponding compound and protein. The best-docked pose was visualized and analyzed to explore the non-bonded interactions using PyMOL Molecular Graphics System 2.0 (DeLano Scientific LLC, San Carlos, CA, USA), UCSF Chimera 1.12 (RBVI, University of California, San Francisco, CA, USA), and Discovery Studio 4.5 (Accelrys, San Diego, CA, USA).

### 3.10. Calculation of Pharmacokinetic Parameters

The drug likeness properties of the compounds were predicted using the Molinspiration online toolkit (http://www.molinspiration.com/cgi-bin/properties). Orally active drugs should comply with these widely utilized drug likeness properties to prove their pharmaceutical fidelity. In this study, molecular descriptors such as miLogP, the number of hydrogen-bond donors, the number of hydrogen-bond acceptors, the molecular mass of the compounds, TPSA, the number of rotatable bonds, and violations of Lipinski’s rule of five [33] were calculated. According to a previously described method [35], absorption (% ABS) was calculated using the following formula:

% ABS = 109 − (0.345 × TPSA).

### 3.11. Prediction of Toxicological Properties

Since toxicity is a major concern for using any drug, we predicted toxicological properties of the compounds with the admetSAR online toolkit (http://lmmd.ecust.edu.cn:8000/), which is reported to be an important and useful predictor in drug discovery [50]. Ames toxicity, carcinogenic properties, acute oral toxicity, rat acute toxicity, and inhibitory effects on hERG were predicted, and the results are summarized in Table 4.

### 3.12. Density Functional Theory (DFT) Calculation

All compounds were fully optimized by DFT employing Becke’s three-parameter hybrid model, Lee–Yang–Parr (B3LYP) correlation functional method at the 6–31G (d,p) level, using Gaussian09 software [47]. Fully optimized structures of the compounds are shown in Figure S2. Electronic energies (E), enthalpy (H), Gibbs free energies (G), dipole moments, and frontier molecular orbital energies were also investigated using the same level of theory. HOMO–LUMO gaps of each compound were calculated by subtracting the LUMO energy value from the corresponding HOMO energy value of the compound. The hardness (*η*) and softness (*S*) of each compound were calculated from the energies of frontier HOMO and LUMO according to the following equations:

*η* = [*ε*HOMO − *ε*LUMO]/2; *S* = 1/*η*.

### 3.13. Statistical Analysis

Data are expressed as means ± standard error (SE). Results were analyzed using a Student’s *t*-test with IBM SPSS Statistics 24 (IBM Corporation, Armonk, NY, USA). Values of *p* < 0.05 and *p* < 0.01 were considered statistically significant.

## 4. Conclusions

In summary, we found that the prenylated flavonoids of OP significantly inhibited albumin denaturation in vitro, demonstrating their protective effects on cellular proteins from destruction due to chronic inflammation. Moreover, OP compounds reduced nitrite accumulation and COX-2 activity significantly in an inflammatory model of LPS-stimulated RAW 264.7 cells. All compounds inhibited α-glucosidase and acetylcholinesterase enzyme activity in a dose-dependent fashion, which correlate with the findings of their molecular docking simulations. They could strongly bind to the active site or close allosteric sites of α-glucosidase and acetylcholinesterase protein receptors. Pharmacokinetics and toxicological properties revealed their drug-like properties, confirming them as safe drug sources. Global reactivity descriptors determined from DFT-based computations confirmed that OP compounds have a strong reactive nature showing low HOMO–LUMO energy gaps. However, the potential pharmacological properties of OP components as anti-inflammatory, anti-diabetic, and anti-Alzheimer’s drugs described might also be attributed to their PAK1-inhibiting mechanisms reported in our previous findings. Collectively, this investigation allows the conclusion that OP components might be novel and safe drug sources for the treatment of inflammation, diabetes, and AD.

## Figures and Tables

**Table 1:** Half maximal inhibitory concentration (IC_50_) values of nitrite inhibition by different compounds. NA—nymphaeol-A; NB—nymphaeol-B; NC—nymphaeol-C; INB—isonymphaeol-B; GN—3′-geranyl-naringenin.

Compound	Nitrite Inhibition (IC_50_ Value, µM)
NA	3.2
NB	5.4
NC	2.4
INB	6.2
GN	7.0

**Table 2:** Binding affinity and binding interactions of Okinawa propolis (OP) compounds with α-glucosidase (Protein Data Bank identifier (PDB ID): 3A4A) and acetylcholinesterase (PDB ID: 4EY7) proteins.

Compound	Binding Affinity (kcal/mol)	Hydrogen Bonds	Hydrophobic Bonds					Electrostatic Bonds
			π–σ	π–π Stacked	π–Alkyl	Alkyl	Amide–π Stacked	
**3A4A**								
NA	−9.3	Asp^352^ (1.92)	Tyr^158^ (3.56)		Arg^315^ (4.88) Tyr^158^ (4.11) Phe^314^ (4.97)	Lys^156^ (5.05)		Arg^442^ (4.67)
NB	−8.7	Gln^353^ (2.20) Arg^315^ (3.50)	Arg^315^ (3.98)	Phe^303^ (4.62)	Tyr^158^ (4.36) Phe^314^ (5.10) Phe^314^ (4.89)	Arg^315^ (5.18) Arg^315^ (4.42)		
NC	−9.9	Ser^311^ (2.82) Asp^352^ (2.17)	Phe^178^ (3.71)		Arg^315^ (4.51) Tyr^72^ (4.51) His^112^ (4.90) Phe^314^ (5.00) Phe^314^ (4.96) Tyr^316^ (5.02)	Arg^315^ (4.36) Arg^315^ (4.37) Val^216^ (4.33)	Phe^314^ (5.83) Arg^315^ (5.83)	
INB	−9.2	Glu^277^ (2.24) Asp^352^ (2.60) Arg^315^ (2.21)			Arg^315^ (3.85) Tyr^158^ (4.73) His^280^ (5.32)	Lys^156^ (3.47) Lys^156^ (4.03)		Arg^442^ (4.07)
GN	−7.1	Lys^466^ (2.44) Pro^467^ (2.31) Trp^36^ (2.19)		Trp^36^ (5.38)	Phe^469^ (4.97) Tyr^470^ (4.63)			
**4EY7**								
NA	−11.5		Trp^86^ (3.97)	Tyr^337^ (5.41) Tyr^341^ (4.00)	Trp^86^ (4.05) Tyr^124^ (4.93) Phe^297^ (5.23) Tyr^337^ (3.95) Phe^338^ (4.79) His^447^ (5.43) His^447^ (4.46)			
NB	−11.2	Asp^74^ (2.02) Arg^296^ (1.86) Tyr^72^ (2.05) Thr^75^ (2.77) Phe^295^ (2.77)	Tyr^341^ (3.67)		Leu^76^ (4.61) Trp^86^ (4.57) Tyr^337^ (4.02) Tyr^337^ (4.52) Phe^338^ (5.36) His^447^ (4.60)			
NC	−11.0	Ser^293^ (2.17) Tyr^72^ (2.07) Tyr^72^ (2.02) Phe^295^ (2.69) Arg^296^ (2.31) Arg^296^ (2.29)	Tyr^341^ (3.73)		Leu^76^ (4.86) Trp^86^ (4.63) Tyr^337^ (4.41) Tyr^337^ (4.02) Phe^338^ (5.29) His^447^ (4.57)			
INB	−11.0	Asp^74^ (2.00) Phe^295^ (1.88)	Trp^86^ (3.89)	Tyr^337^ (5.10) Trp^286^ (4.01) Tyr^341^ (4.72) Trp^286^ (5.32)	Trp^86^ (4.57) Trp^86^ (5.07) Tyr^337^ (5.39) His^447^ (4.71)			
GN	−11.3	Gln^291^ (2.29) Tyr^124^ (1.89)	Tyr^341^ (3.75) Trp^86^ (3.78) Trp^86^ (3.88) Trp^86^ (3.94)	Trp^286^ (3.92) Trp^286^ (4.98)	Trp^86^ (5.04) Tyr^124^ (5.35) Phe^297^ (5.36) Tyr^337^ (4.91) Phe^338^ (4.64)			

Values in the bracket indicate the bond distance (Å).

**Table 3:** Physicochemical properties of the compounds for good oral bioavailability.

Compound	% ABS ^a^	TPSA (Å^2^) ^b^	MW ^c^	MiLogP ^d^	HBD ^e^	HBA ^f^	n-ROTB ^g^	Lipinski’s Violation
**Rule**	-	-	˂500	≤5	˂5	˂10	≤10	≤1
NA	72.01	107.22	424.49	5.52	4	6	6	1
NB	72.01	107.22	424.49	5.49	4	6	6	1
NC	72.01	107.22	492.61	7.53	4	6	8	1
INB	72.01	107.22	424.49	5.49	4	6	6	1
GN	78.99	86.99	408.49	6.21	3	5	6	1

^a^ Percentage of absorption; ^b^ topological polar surface area; ^c^ molecular weight; ^d^ logarithm of compound partition coefficient between *n*-octanol and water; ^e^ number of hydrogen-bond donors; ^f^ number of hydrogen-bond acceptors; ^g^ number of rotatable bonds.

**Table 4:** Toxicological properties of the compounds.

Parameters	Compound				
	NA	NB	NC	INB	GN
Ames toxicity	Non Ames toxic	Non Ames toxic	Non Ames toxic	Non Ames toxic	Non Ames toxic
Carcinogens	Non-carcinogenic	Non-carcinogenic	Non-carcinogenic	Non-carcinogenic	Non-carcinogenic
Acute oral toxicity	III	III	III	III	III
Rat acute toxicity	3.1399	3.1399	3.1399	3.1399	3.1399
hERG ^a^	Weak inhibitor	Weak inhibitor	Weak inhibitor	Weak inhibitor	Weak inhibitor
Carcinogenicity (Three-class)	Not required	Not required	Not required	Not required	Not required

^a^ Human ether-a-go-go-related gene.

**Table 5:** Results of quantum chemical calculations and thermodynamic properties of the compounds.

Compound	Electronic Energy (Hartree)	Enthalpy (Hartree)	Gibbs Free Energy (Hartree)	Dipole Moment (Debye)	εHOMO (Hartree)	εLUMO (Hartree)	Gap (Hartree)	*η*	*S*
NA	−1420.38	−1420.38	−1420.48	3.337	−0.2097	−0.0465	0.1632	0.08161	12.2526
NB	−1420.37	−1420.37	−1420.47	3.168	−0.2107	−0.0502	0.1605	0.08028	12.4564
NC	−1615.60	−1615.60	−1615.72	5.228	−0.2090	−0.0489	0.1600	0.08004	12.4929
INB	−1420.38	−1420.38	−1420.47	2.235	−0.2135	−0.0536	0.1599	0.07995	12.5078
GN	−1345.16	−1345.16	−1345.25	4.959	−0.2207	−0.0522	0.1684	0.08423	11.8715

εHOMO = highest occupied molecular orbital energy; εLUMO = lowest unoccupied molecular orbital energy; *η* = hardness; *S* = softness.

## Supplementary Materials

The following are available online, Figure S1: Visualization of frontier molecular orbitals of Okinawa propolis (OP) compounds. (A,B)—HOMO and LUMO of nymphaeol A (NA); (C,D)—HOMO and LUMO of nymphaeol B (NB); (E,F)—HOMO and LUMO of nymphaeol C (NC); (G,H)—HOMO and LUMO of isonymphaeol B (INB); (I,J)—HOMO and LUMO of 3′-geranyl-naringenin (GN); Figure S2: Optimized structures of OP compounds. (A)—NA, (B)—NB, (C)—NC, (D)—INB, (F)—GN.
