# Supplementary material for: Anti-Inflammatory, Anti-Diabetic, and Anti-Alzheimer’s Effects of Prenylated Flavonoids from Okinawa Propolis: An Investigation by Experimental and Computational Studies
Source: Molecules. 2018 Sep 27;23(10):2479. doi: 10.3390/molecules23102479 (PMC6222853; doi:10.3390/molecules23102479)
Supplement: Supplementary file 1 [file molecules-23-02479-s001.pdf]

Supporting information

Anti-Inflammatory, Anti-Diabetic, and Anti-Alzheimer's Effects of Prenylated Flavonoids from Okinawa Propolis: An Investigation by Experimental and Computational Studies

Md Shahinozzaman [1,2], Nozomi Taira [3], Takahiro Ishii [1,3], Mohammad A. Halim [4], Md Amzad Hossain [1,5] and Shinkichi Tawata [2,3,*]

[1] The United Graduate School of Agricultural Sciences, Kagoshima University, Korimoto 1-21-24, Kagoshima 890-0065, Japan; mshahin81@gmail.com (M.S.); ishiit@agr.u-ryukyu.ac.jp (T.I.); amzad@agr.u-ryukyu.ac.jp (M.A.H.)

[2] PAK Research Center, University of the Ryukyus, Okinawa 903-0213, Japan

[3] Department of Bioscience and Biotechnology, Faculty of Agriculture, University of the Ryukyus, Senbaru 1, Nishihara-cho, Okinawa 903-0213, Japan; taira5935@gmail.com

[4] Division of Computer Aided Drug Design, The Red-Green Research Centre, 218 Elephant Road, Dhaka-1205, Bangladesh; mahalim@grc-bd.org

[5] Subtropical Field Science Center, University of the Ryukyus, Senbaru 1, Nishihara-cho, Okinawa 903-0213, Japan

* Correspondence: b986097@agr.u-ryukyu.ac.jp; Tel.: +81-090-5472-6883

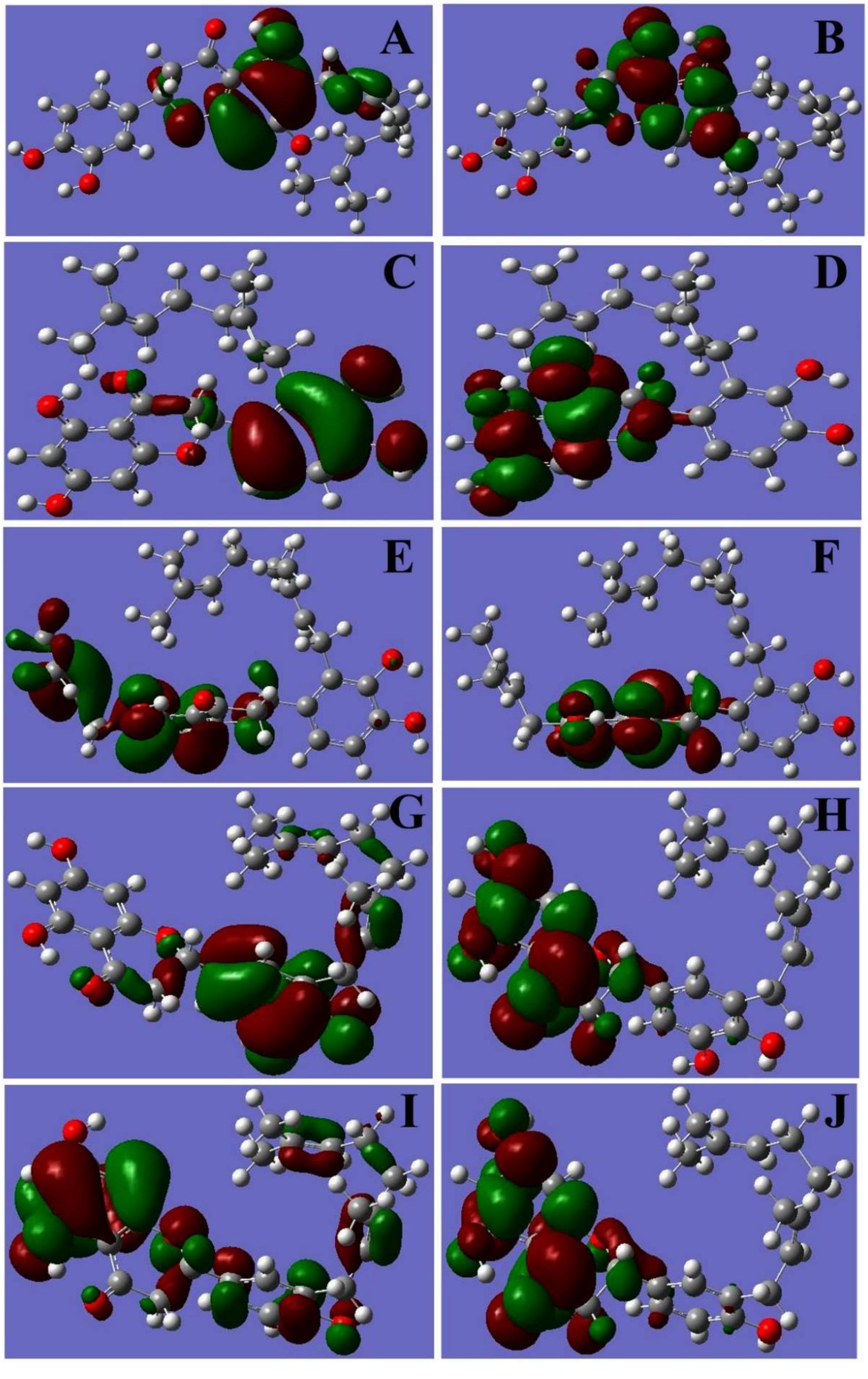

Figure S1. Visualization of frontier molecular orbitals of Okinawa propolis (OP) compounds. A, B – HOMO and LUMO of nymphaeol A (NA); C, D – HOMO and LUMO of nymphaeol B (NB); E, F – HOMO and LUMO of nymphaeol C (NC); G, H – HOMO and LUMO of isonymphaeol B (INB); I, J – HOMO and LUMO of 3′-geranyl-naringenin (GN). HOMO = highest occupied molecular orbital; LUMO = lowest unoccupied molecular orbital

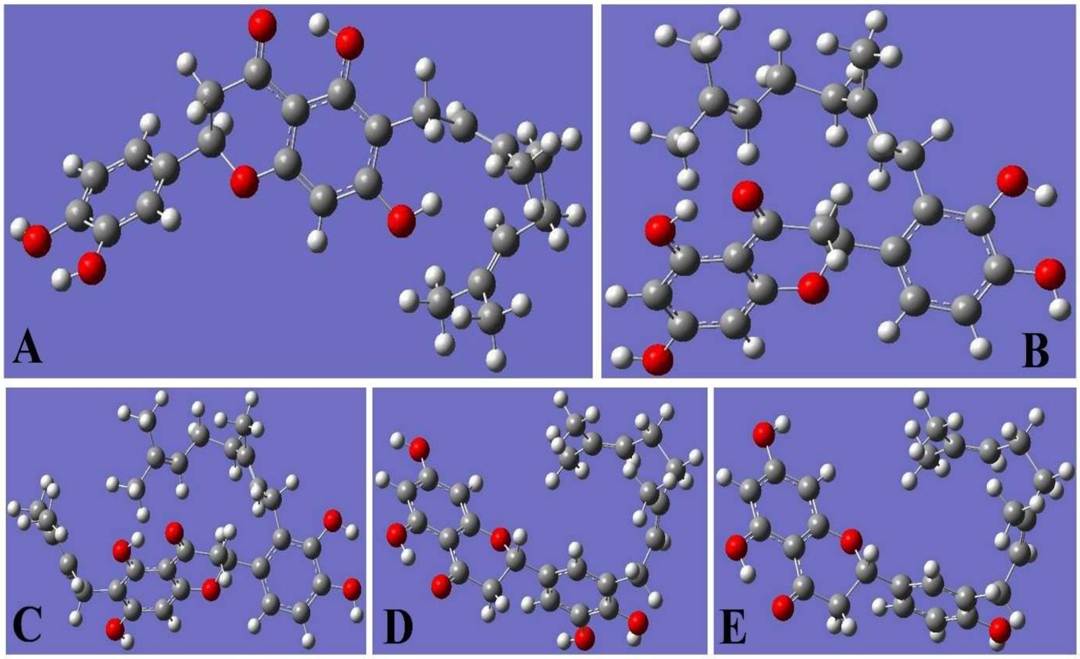

Figure S2. Optimized structures of OP compounds. A – NA, B – NB, C – NC, D – INB, F – GN.
